# Research and Application Validation of a Feature Wavelength Selection Method Based on Acousto-Optic Tunable Filter (AOTF) and Automatic Machine Learning (AutoML)

**DOI:** 10.3390/ma15082826

**Published:** 2022-04-12

**Authors:** Zhongpeng Ji, Zhiping He, Yuhua Gui, Jinning Li, Yongjian Tan, Bing Wu, Rui Xu, Jianyu Wang

**Affiliations:** 1 Key Laboratory of Space Active Opto-Electronics Technology, Shanghai Institute of Technical Physics of the Chinese Academy of Sciences, Shanghai 200083, China; jizhongpeng@aliyun.com (Z.J.); yhgui@mail.ustc.edu.cn (Y.G.); lijinning@mail.sitp.ac.cn (J.L.); tanyongjian@mail.sitp.ac.cn (Y.T.); wubing@mail.sitp.ac.cn (B.W.); xurui@mail.sitp.ac.cn (R.X.); 2 University of Chinese Academy of Sciences, Beijing 100049, China

**Keywords:** AOTF, AutoML, feature wavelength selection, near infrared detection system

## Abstract

Near-infrared spectroscopy has been widely applied in various fields such as food analysis and agricultural testing. However, the conventional method of scanning the full spectrum of the sample and then invoking the model to analyze and predict results has a large amount of collected data, redundant information, slow acquisition speed, and high model complexity. This paper proposes a feature wavelength selection approach based on acousto-optical tunable filter (AOTF) spectroscopy and automatic machine learning (AutoML). Based on the programmable selection of sub nm center wavelengths achieved by the AOTF, it is capable of rapid acquisition of combinations of feature wavelengths of samples selected using AutoML algorithms, enabling the rapid output of target substance detection results in the field. The experimental setup was designed and application validation experiments were carried out to verify that the method could significantly reduce the number of NIR sampling points, increase the sampling speed, and improve the accuracy and predictability of NIR data models while simplifying the modelling process and broadening the application scenarios.

## 1. Introduction

NIR spectroscopy has many advantages, such as being nondestructive and accurate, and has been widely applied in areas such as food safety [1,2], drug analysis [3], agricultural testing [4], and basic chemistry [5]. The current common approach to NIR spectroscopy is to obtain the full continuous spectral data of the sample in the spectral range and to use the corresponding algorithms to model the correlation between the sample and the spectral data. However, the NIR spectral data of a sample has a relatively high dimensionality and also suffers from inter-spectral overlap, covariance, and noise, which negatively affects the performance of the NIR spectral model [6]. The selection of the effective feature wavelengths of the sample is extremely important at this point. Feature wave-length selection extracts spectrally valid variables and removes useless or interfering wavelength data, improving the accuracy and predictiveness of the data model. Only spectral data from a specific band or specific wavelength points are required to build a well-performing detection model, requiring significantly fewer wavelength sampling points.

Numerous algorithms exist to select the characteristic wavelengths of the collected NIR spectra of the samples to build reliable models [7], such as the adaptive reweighted sampling (CARS) [8], the random frog hopping algorithm (RF) [9], the PLS–genetic algorithm (PLS-GA) [10], etc. These algorithms have different performance for different data and problems, and in practice modelling usually involves human experience in selecting the most suitable model, which increases the complexity of the modelling process. In contrast, the recently proposed automatic machine learning (AutoML) [11] allows for automated model selection without human intervention. The model automatically generates the network structure that is most efficient for the task at hand, and the model automatically searches for the best sequence of combinations of operations under the different structures produced by itself. This approach can effectively reduce the complexity of modelling and also ensures the robustness of the model.

After modelling the characteristic wavelengths of the sample, the current instruments still need to collect the full spectrum first and then select the characteristic spectra to input into the model to obtain the final results [12,13]. However, this full-spectrum acquisition method results in slow data acquisition and processing. Although common grating-based spectroscopy instruments can acquire data quickly, the cost is significantly higher due to the use of array detectors, and problems such as non-uniformity between detectors can also affect the signal-to-noise ratio of the acquisition. In some applications where fast real-time processing is required, such as industrial on-line analysis, faster spectral acquisition and higher data quality are often required. Therefore, an NIR spectrometer that can be coupled with a feature wavelength filtering algorithm to achieve variable wavelength acquisition is a solution that can guarantee both speed and data quality and model robustness. An AOTF spectrometer can change the diffraction wavelength by changing the frequency of the RF power signal added to it, which can achieve sub-nm-level central wavelength picking in the full spectrum and use a unit detector to obtain data, which can effectively improve the signal-to-noise ratio and is an ideal device that can be used with the feature wavelength selection algorithm.

AOTF-based NIR spectroscopy has been extensively studied in food inspection and agricultural applications. Several studies have been conducted to implement commercial AOTF-NIR spectrometers for nondestructive detection of dried apple and olive fruit [14,15,16,17]. Diffuse reflectance spectra were acquired in the wavelength range 1100–2300 nm at 2 nm intervals using the Luminar 5030 AOTF-NIR Miniature ‘Hand-held’ Analyzer (Brimrose Corporation, Baltimore, MD, USA). The feasibility of using AOTF-NIR spectroscopy in an intelligent drying system for nondestructive detection and monitoring of physicochemical changes in organic apple wedges during the drying process was investigated. Partial least squares (PLS) regression models were developed to monitor changes in water activity, moisture content, soluble solids content, and chroma during drying. The classification models were computed using K-means and Partial Least Squares Discriminant Analysis (PLS-DA) algorithms in sequence [14]. AOTF-NIR was also satisfactorily applied to predict phenolic compounds [15] and monitor the ripening of olives [16]. Water content, fat content, and free acidity in olive fruit were predicted by online NIR spectroscopy combined with chemometrics techniques [17]. In addition to the PLS regression algorithm, a sensor software based on an artificial neural network (SS-ANN) was designed by Allouche et al. [18] for monitoring olive malaxation. In hyperspectral imaging, a prototype on-line AOTF-based hyperspectral image acquisition system (450–900 nm) has also been developed for tenderness assessment of beef carcasses [19]. However, in these previous studies, it is usually necessary to collect all spectral data before making predictions, and for AOTF spectroscopy more research is needed to optimize the sampling strategy according to specific applications.

Therefore, this paper utilizes the features of AOTF programming to acquire specific wavelengths and combines an AOTF-based spectrometer with an automatic machine learning–based feature wavelength selection algorithm to achieve rapid output of target substance detection results in the field. This form of application will effectively improve the efficiency of online NIR spectroscopy systems and bring broader prospects for industry applications, as it can significantly reduce the amount of data to be collected and reduce the time required for the cyclic data collection process during online inspection.

## 2. AutoML-Based Feature Wavelength Selection

**Figure 1:**
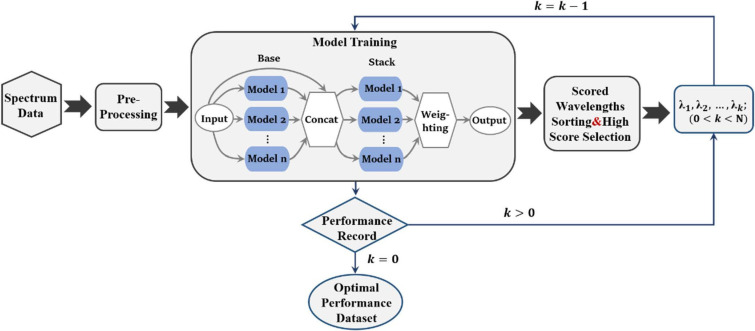
Feature wavelength selection using the AutoGluon-Tabular architecture.

The efficiency of current machine learning algorithms often relies on human guidance, such as data preprocessing, feature selection, algorithmic models, and the determination of hyperparameters. With the complexity of machine learning algorithms, the number of available algorithms and processes is increasing. AutoML is a spatial search optimization method that can find the optimal solution in a finite spatial range in the shortest possible time, reducing time and labor costs while improving operational accuracy [11]. AutoML generally consists of two main components: an evaluator and a tuner. The evaluator is responsible for measuring the performance of the learning tool under the adoption number conditions provided by the tuner and for feeding the results to the tuner, while the tuner is responsible for making the feedback information for the learning tool to update the configuration information. In addition to algorithm selection, hyperparameter optimization [20], and neural network architecture searches, AutoML can also cover automatic data preparation, automatic feature selection, automatic flowline construction, automatic model selection, and integrated learning [21]. One of the solutions for AutoML is AutoGluon–Tabular architecture [22], which is very suitable for structured data regression problem solving with more accurate model robustness. The analytical modeling of NIR spectral data is a typical structured data regression problem, and the screening of feature wavelengths is also a variable selection problem in machine learning, so it is very suitable for AutoGluon–Tabular architecture, which is used in this paper for the analytical modeling and model-based feature wavelength screening of NIR spectral data. This architecture differs from the common hyperparameter search-based technique architecture in that it relies on fusing multiple models that do not require a hyperparameter search, thus avoiding hyperparameter search and increasing the number of trained models in a prescribed time. The operational steps and the flow chart for data feature wavelength selection of NIR spectral data using AutoGluon–Tabular architecture are as shown in Figure 1.

(1) Data pre-processing

Firstly, the acquired transmission/reflection spectral data are converted into absorbance data, and the data are loaded into AutoGluon–Tabular. AutoGluon–Tabular will automatically check the label columns, determine the type of problem, and distinguish whether it is a classification problem or a regression problem; then the data are pre-processed, the feature data are classified, and the useless feature data are discarded.

(2) Model training

After the data pre-processing, the data will be trained by a series of machine learning models, using integration and stacking techniques to combine multiple models. AutoGluon–Tabular will select models for training in a unique order, firstly selecting the models with reliable performance and then gradually selecting the more computationally intensive but less reliable models. The current AutoGluon–Tabular architecture supports algorithms such as Random Forest [23], Super Random Tree, k Nearest Neighbor, LightGBM boosted tree, CatBoost boosted tree, AutoGluon–Tabular deep neural network, etc. In this paper, we will train all of the above algorithms. AutoGluon–Tabular ensembles multiple models and stacks them in multiple layers, which offers better use of allocated training time than seeking out the best. We set ‘root_mean_squared_error’ as “eval_metric”. AutoGluon–Tabular tunes factors such as hyperparameters, early-stopping, ensemble-weights, etc. in order to improve this metric on validation data.

(3) Wavelength importance ranking

We calculated the permutation importance which measures the importance of a feature and ranked the wavelength variables according to this. The higher the permutation importance, the higher the contribution of the wavelength to the model and the more representative the wavelength is of the characteristics of the detection target.

(4) Feature wavelength combination screening

After ranking the importance of wavelengths, we are not sure which wavelength combinations can achieve the best performance of the model. In order to filter the best combination of feature wavelengths, we select the wavelengths with the top X permutation importance to retrain the model, record the evaluation index of the model performance trained by X wavelengths, reduce the number of X by 1, and repeat the retraining process until X equals 0, then stop the training. Based on the number of different X’s and the evaluation metrics of the model performance, we introduce the precision criterion to determine the optimal number of wavelength variables. If a precision value is entered, the model suggested according to the precision criterion is the model with the lowest number of variables among all models, whose evaluation metric differs from the minimum evaluation metric by no more than the precision.

Finally, we determined the optimal wavelength combination to achieve the minimum number of measurements for a more effective model.

## 3. Instrument Design and Working Principle

After filtering the feature wavelengths of spectral data by AutoML, the construction of the excellent performance model and the accurate prediction results can be achieved via the collection of spectral data at specific wavelengths; however, the extraction of the feature wavelengths of the current conventional spectrometer spectral data requires us to obtain the full-band spectrum by scanning in advance, which results in sampling time waste and resource consumption. Thus, the above problems can be effectively solved if the wavelength filtering results can be targeted and finely sampled, while the AOTF near-infrared spectrometer has the characteristic of freely adjustable wavelength, which allows it to conduct discrete sampling and perform targeted and finely spectral sampling for specific filtering results.

**Figure 2:**
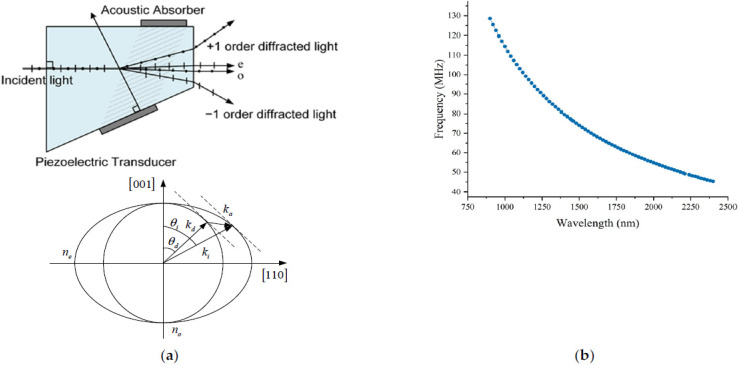
AOTF spectroscopy schematic. (**a**) AOTF spectroscopy and wave vector diagram; (**b**) curve of AOTF driving frequency with diffraction wavelength.

AOTF is an electrically tunable filter based on the acousto-optic effect, which is mainly composed of a birefringent acousto-optic crystal (the most widely used material TeO_2_), a piezoelectric transducer, and an acoustic wave absorber, as shown in Figure 2a. In practical applications, the RF drive signal is converted to ultrasonic waves inside the crystal by a piezoelectric transducer fixed on the crystal surface, and the diffraction wavelengths are selected by changing the frequency of the RF signal, with a wide tuning range and fast scanning speed. Spectrometers based on AOTF spectroscopy have the advantages of small size, light weight, all-solid state, and strong environmental adaptability and have been widely used in many fields such as food inspection [24], environmental monitoring [25,26], and deep space exploration [27,28]. According to the condition of momentum matching, the tuning relationship between the driving frequency f(λ) of AOTF and the output diffraction wavelengths is shown as Equation (1) [29,30]:

(1) $$f_{a}\left(\lambda \right)=V_{a}\left[n_{i}^{2}+n_{d}^{2}-2n_{i}n_{d}\cos(\theta_{i}-\theta_{d}\right){]}^{1/2}/\lambda ,$$

where λ is the incident light wavelength, $V_{a}$ is the ultrasonic propagation velocity in the crystal, $f_{a}\left(\lambda \right)$ is the corresponding ultrasonic frequency, $\theta_{i}$ is the incident angle for a device, $\theta_{d}$ is the output beam angle, $n_{i}$ is the refractive index for the incident light, and $n_{d}$ is the refractive index for the diffracted light. The relationship curve between the diffraction wavelength of the crystal and the driving frequency is shown in Figure 2b. The curve data were obtained by measurement. A HORIBA iHR 320 spectrometer was used to generate monochromatic light. The wavelength of the monochromatic light was first set, and then the scanning was performed by changing the driving frequency in fine steps. The frequency at which the most intense diffracted light was measured is the driving frequency corresponding to that wavelength. This procedure was repeated for the entire operating spectral range to obtain all data. It is possible to realize both sequences in the time-fine scanning of wide spectrum bands by wavelength point by point and the high-precision diffraction center wavelength picking of specific wavelength bands by coding control of RF driving frequency when using a good correspondence between RF driving frequency and diffraction wavelength, with simple wavelength picking and good repeatability.

**Figure 3:**
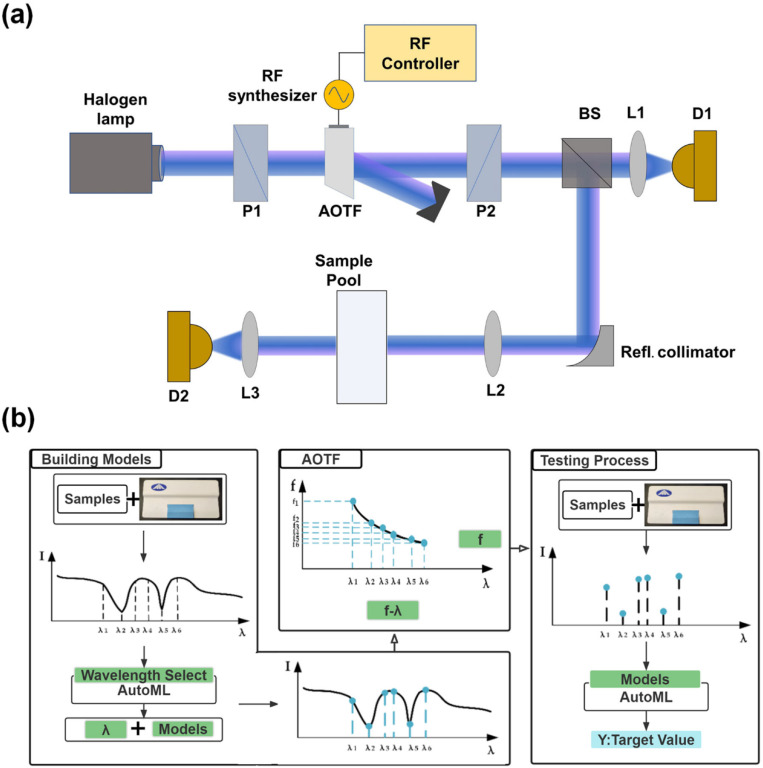
AOTF detection system and testing process. (**a**) schematic diagram of AOTF dual optical path and dual detector NIR spectrometer; P1, P2, polarizer; BS, adjustable beam splitter; D1, D2, InGaAs photodetector; (**b**) flow chart of AOTF detection based on AutoML.

According to the characteristics of the AOTF spectrometer, the optical path structure of this system is shown in Figure 3a by combining an AutoML autonomous feature wavelength filtering algorithm and AOTF spectrometer-specific band sampling. The use of a dual optical path and a dual detector design, the use of an adjustable beam splitter to introduce the reference optical path, comprehensive data processing, and compensation of light intensity fluctuations caused by the instability of the light source and errors caused by environmental interference (in order to improve the accuracy of the instrument) allowed us to achieve a wide spectral scan range of 900–2400 nm. The actual spectra are sampled by a short-wave infrared AOTF, using both high-frequency and low-frequency drivers to achieve a wide spectral sampling range of 900–2400 nm, with a spectral resolution of 3.75–9.6 nm. The main performance parameters of the AOTF are shown in Table 1.

The flow chart of the system is shown in Figure 3b. Firstly, the full spectrum data of the substance is obtained through the AOTF spectrometer, and the filter modeling of the feature wavelength of the target is completed using the AutoML algorithm to extract the feature wavelength of the sample. After that, the RF drive function of the feature wavelengths is generated using the selected feature wavelength combinations combined with the drive frequency function of AOTF, and the drive function is stored in the RF controller. When the target is measured again, the system can take advantage of the flexible adjustment of the AOTF central wavelength at the subnanometer level to accurately locate the desired band and achieve the adjustable, high-precision, and fast acquisition of the feature spectrum. By transferring the obtained data to the model established by the combination of feature wavelengths, the detection and analysis of the characteristic components or contents of the sample can be realized.

## 4. Application Results

To verify the effectiveness of the system, two different types of liquid samples (milk and baijiu) were selected, and validation experiments were performed according to the detection system and testing process shown in Figure 3. The experimental results of the two measurement methods for fat content (%) in milk samples and alcohol by volume (ABV, %) in baijiu samples are shown.

### 4.1. Sample Preparation

For the milk samples, we chose nine different brands of liquid pure milk, and five samples of each milk brand were prepared for a total of 45 samples. The nine milk samples had different fat contents, as shown in Table 2, to establish the relationship between spectral data and fat content in the milk model using the nominal value of fat content.

For the liquor samples, we selected two brands and five types of liquor divided into 209 samples marked and sealed for storage. The determination of alcoholic content was entrusted to the Shenzhen Institute of Measurement and Quality Inspection using the national standard (GB 5009.225-2016) alcoholometer method, and the measurement result was the volume fraction of alcohol, i.e., alcoholic content (%vol). The range of alcoholic content of the samples was 42.0–56.2, with an accuracy error of 0.1, as shown in Table 3.

The NIR transmittance spectra of all samples were collected using this system with a spectral sampling range of 900–2400 nm and a sampling interval of 5 nm, and the NIR spectral data for each sample was 301 wavelength points. For the milk samples, a 0.5 mm optical path length quartz cuvette was used to hold the samples, and the transmittance spectra of 45 samples were obtained. For the baijiu samples, the transmittance spectra of 209 samples were obtained using a quartz cuvette with a 2 mm optical path length.

**Figure 4:**
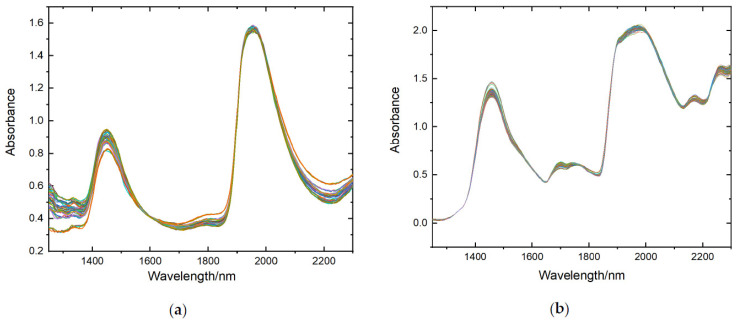
Absorbance spectra of different samples. (**a**) milk samples; (**b**) alcohol samples.

The absorbance spectra of all samples were obtained by taking log(1/T) of the transmittance spectra of all samples. Savitzky-Golay (SG) [31,32] smoothing was used to reduce the random noise. The absorbance spectra were processed using Multiplicative Scatter Correction (MSC) [33] to remove the influence of non-concentration factors such as baseline shift and granularity on the spectra. The spectral data with low signal-to-noise ratio at the head and tail were removed, and the final data were in the wavelength range of 1250–2300 nm and 5 nm sampling interval, with a total of 211 data points. The absorbance spectral data obtained by processing are shown in Figure 4.

### 4.2. Modeling and Feature Wavelength Selection

The spectra of milk and alcohol samples obtained by AOTF spectrometer were converted into spectral absorbance values as input features, and fat content and alcohol content were used as model target values. The data sets were randomly divided, with 70% (31 samples) of milk used as training data and the remaining 30% (14 samples) used as test data; 70% (146 samples) of baijiu was used as training data and the remaining 30% (63 samples) was used as test data, as shown in Table 4 and Table 5. Only the training data was provided to AutoML framework at training time, while the test data was only provided at prediction time.

The fit application programming interface (API) of AutoGluon–Tabular was used to train these models. Within the call to fit, AutoGluon automatically preprocesses the raw data, identifies what type of prediction problem this is (binary, multi-class classification, or regression), partitions the data into various folds for model-training vs. validation, individually fits various models, and finally creates an optimized model ensemble that outperforms any of the individual trained models.

**Figure 5:**
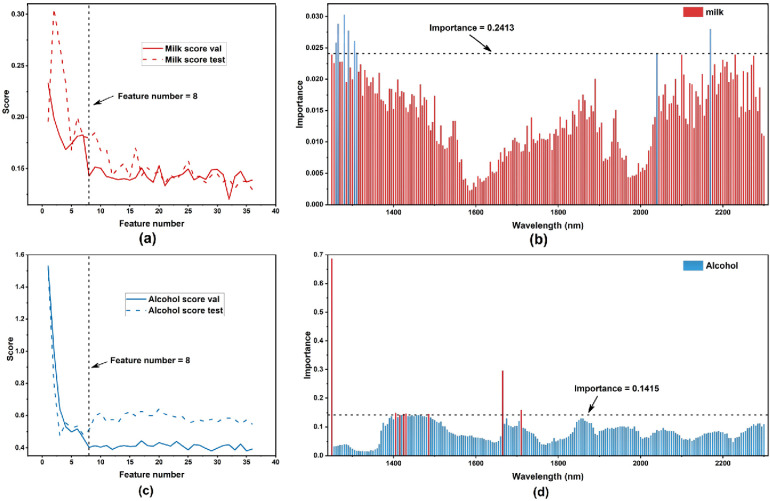
Results of the “score val” (RMSEV) and permutation importance of the two samples. (**a**) “score val” (RMSEV) of milk samples. We also calculated RMSEP as “score test”. (**b**) degrees of permutation importance of the milk samples; (**c**) “score val” (RMSEV) of alcohol samples. We also calculated RMSEP as “score test”. (**d**) permutation importance of alcohol samples.

The performance of these models was adjudged based on root mean square errors (RMSE) of validation (RMSEV) and prediction (RMSEP). RMSEV is obtained from AutoGluon which is named as “score val,” and RMSEP is calculated based on the prediction results of the test set. This is as shown in Figure 5a,c.

The importance the ranking [34] of data variables was performed based on permutation importance for milk and baijiu samples, as shown in Figure 5b,d. Higher importance indicates that the feature variables have more influence on the model performance.

Based on the accuracy criteria, we calculated the difference between the RMSEV and the minimum RMSEV for all models, where the model with a difference not higher than 20% and with the least number of variables was selected. According to our precision criterion, eight characteristic wavelengths were selected for milk, and eight characteristic wavelengths were selected for baijiu. The actual results of the selected wavelengths are shown in Table 6.

### 4.3. Experimental Results of the System

The AOTF-NIR spectroscopy system combining the model and the corresponding sampling strategy was deployed to a real application scenario to perform experiments on the measurement of samples with unknown target value content. Using this instrumentation system with models, we were able to perform 211-point and 8-point measurements on the samples, respectively. The data obtained from the measurements could then be used to invoke the corresponding model for component content prediction. The analytical performance of the system was evaluated when the full-spectrum model and the characteristic wavelength model were applied separately. The performance of the prediction could be evaluated by calculating new RMSEPs.

The RMSEV shown in Table 7 is the “score val” obtained during the training phase. We also performed 211-point and 8-point spectral acquisitions for the above test set of samples, respectively. After data acquisition, the average spectrum calculated in the model building phase is used as the true spectrum for MSC processing. No smoothing operation is performed at this stage. The data are then input to the model to derive predicted values, which are displayed on the software interface. The RMSEP metric is obtained by calculating the error between the predicted and true values obtained from the inference of the input model after the spectral measurement; the inference time is automatically recorded by the program in the host computer; the sampling time is the estimated time taken by the instrument to acquire data at the desired wavelength point.

As shown in Table 7 above, the use of the characteristic wavelength model and the corresponding sampling strategy achieves a slight improvement in prediction performance, while the sampling time consumption is ~22-fold smaller. The prediction performance improvement indicates that there are many irrelevant wavelengths throughout the spectrum that do not contribute to effective prediction of the target values and negatively affect the performance of the prediction model.

Combined with the above experimental results, the automatic machine learning framework can accurately and robustly filter the wavelength combinations with the best prediction performance for the target values; combined with the eight-wavelength models and the corresponding new sampling mechanism that only collects data at the eight wavelength points, higher prediction performance and considerable improvement in detection efficiency can be obtained compared with the full-spectrum model and the full-spectrum sampling mechanism. This advantage is particularly critical for the application of AOTF-NIR analysis systems for online inspection. The combination of AOTF’s flexibility and automatic machine learning provides a good reference case for the application of intelligent optical inspection in automated production by applying an automatic machine learning framework to easily build a well-performing model for different application objectives and adaptively change the sampling strategy.

## 5. Conclusions

In this study, an NIR detection system based on AOTF with AutoML feature wavelength screening was proposed and validated. Combining various chemometric algorithms, spectral detection based on the characteristic wavelengths instead of the full spectrum achieves the improvement of detection efficiency. Meanwhile, the model is simplified, interpretability is improved, and storage and computational resource usage are reduced. Taking milk and alcohol as examples, the validation experiments achieve a reduction in the number of sampling wavelengths while achieving accurate and nondestructive rapid determination of fat and alcohol content.

## Figures and Tables

**Table 1:** The main performance parameters of the AOTF.

Parameters	
Material	TeO_2_
Spectral coverage/nm	900–2400
FWHM/nm	3.75–8.4 @ < 1380 nm
	4.2–9.6 @ > 1380 nm
RF/MHz	45.25–128.55
Angular aperture/°	>8
Diffraction angle/°	>7.5
Power/W	~2

**Table 2:** The fat contents of the milk samples.

Brand	Number of Samples	Fat (%)
Item1	5	1.6
Item2	5	0
Item3	5	3.5
Item4	5	3.9
Item5	5	3.5
Item6	5	3.0
Item7	5	3.1
Item8	5	3.0
Item9	5	3.1
all	45	--

**Table 3:** ABV of baijiu samples.

Brand	ABV ^1^	Number of Samples	ABV Measured
Item1	42	14	42.0
	50	49	50.2
	53	77	53.1
	56	47	56.2
Item2	56	22	55.8
all	--	209	--

^1^ ABV = alcohol by volume.

**Table 4:** Statistics on fat of milk samples.

Data Sets	Number of Samples	Min	Max	Mean	STD
Total samples	45	0	3.9	2.99	1.12
Calibration set	31	0	3.9	2.96	1.15
Prediction set	14	0	3.9	2.76	1.32

**Table 5:** Statistics on ABV of baijiu samples.

Data Sets	Number of Samples	Min	Max	Mean	STD
Total samples	209	42	56.2	52.66	3.60
Calibration set	146	42	56.2	52.49	3.80
Prediction set	63	42	56.2	53.05	3.07

**Table 6:** Results of feature wavelength selection.

Samples	Selected Wavelengths (nm)
Milk	1280, 1265, 2170, 1290, 1305, 1260, 1310, 2040
Baijiu	1250, 1665, 1710, 1405, 1430, 1425, 1485, 1415

**Table 7:** The performance of the system for two datasets.

Data Sets	Number of Variables	RMSEV	RMSEP	Sampling Duration
milk	211	0.161	0.190	13.3 s
	8	0.143	0.180	0.6 s
baijiu	211	0.529	0.614	13.3 s
	8	0.403	0.507	0.6 s

## Data Availability

Not applicable.
